# Uncovering Urban Temporal Patterns from Geo-Tagged Photography

**DOI:** 10.1371/journal.pone.0165753

**Published:** 2016-12-09

**Authors:** Silvia Paldino, Dániel Kondor, Iva Bojic, Stanislav Sobolevsky, Marta C. González, Carlo Ratti

**Affiliations:** 1 Department of Physics, University of Calabria, Rende CS, Italy; 2 SENSEable City Laboratory, Massachusetts Institute of Technology, Cambridge, MA, United States of America; 3 SENSEable City Laboratory, SMART Centre, Singapore, Singapore; 4 Center for Urban Science + Progress, New York University, Brooklyn, NY, United States of America; 5 Department of Civil and Environmental Engineering, Massachusetts Institute of Technology, Cambridge, MA, United States of America; University of Rijeka, CROATIA

## Abstract

We live in a world where digital trails of different forms of human activities compose big urban data, allowing us to detect many aspects of how people experience the city in which they live or come to visit. In this study we propose to enhance urban planning by taking into a consideration individual preferences using information from an unconventional big data source: dataset of geo-tagged photographs that people take in cities which we then use as a measure of urban attractiveness. We discover and compare a temporal behavior of residents and visitors in ten most photographed cities in the world. Looking at the periodicity in urban attractiveness, the results show that the strongest periodic patterns for visitors are usually weekly or monthly. Moreover, by dividing cities into two groups based on which continent they belong to (i.e., North America or Europe), it can be concluded that unlike European cities, behavior of visitors in the US cities in general is similar to the behavior of their residents. Finally, we apply two indices, called “dilatation attractiveness index” and “dilatation index”, to our dataset which tell us the spatial and temporal attractiveness pulsations in the city. The proposed methodology is not only important for urban planning, but also does support various business and public stakeholder decision processes, concentrated for example around the question how to attract more visitors to the city or estimate the impact of special events organized there.

## Introduction

Outputs of analyses on digital footprints can provide novel insights into how people live and experience the city, revealing important aspects of human mobility including tourism. The most widespread way of extracting information from digital traces is to use mobile phone records [1, 2] which helped scholars to develop accurate methods for understanding human mobility patterns [3–5], land use classification [6–8] or regional delineation [9–11]. Nevertheless, other sources of big data are also becoming increasingly useful, such as digital maps [12], credit card payments [13, 14], online social networks like Twitter [15] or Flickr [16], circulation of bank notes [17], vehicle GPS traces [18], migration networks [19]. In particular, besides Twitter there is a plethora of online social networks, used by a huge number of people every day to share for example their interests, opinions, perceptions, photographs, resulting in the emergence of very large datasets reflecting human behavior.

The focus of this paper is on geo-tagged photographs shared by users through online social media platforms (e.g., Flickr), as publicly shared photographs present a feedback on urban design and planning in a qualitative way and almost in a real time which can be a significant addition to more traditional methods, e.g., surveys [20]. Namely, it has been already shown that the number of geo-tagged photographs taken in a particular city serves as a good proxy for studying city attractiveness [21–24]. Although perhaps they do not always share the same reasons, both residents and visitors take photographs at the places they consider important and that is useful for understanding what people like in cities, what they are interested in or where they like to go. As those insights provide important directions for urban innovations, our goal is not only to measure urban attractiveness at a certain moment or aggregate it over a longer period of time, but also to detect how its patterns change over time.

## Dataset

In our study we use publicly available data from the website sfgeo.org, which collects records about photographs shared on the most popular photograph sharing websites (e.g., Flickr and Picasa) [25]. From this dataset, which contains in total more than 100 million publicly shared geo-tagged photographs taken during a period of 10 years, we omitted duplicates (9.33% of the dataset in total) and photographs with incorrect timestamps (0.01% of the dataset in total). Then, similarly to our previous paper [21], we limited our analysis to only those photographs that were taken between 2007 and 2010 as these compose almost 75% of the entire dataset or about 70 million of photographs in total. Finally, we ordered cities by the number of photographs taken in them (see Table 1) and chose to consider in further analysis ten most photographed ones: New York City, London, Paris, San Francisco, Washington DC, Barcelona, Rome, Chicago, Los Angeles, Berlin.

**Table 1:** Heterogeneity of Flickr usage: total number of photographs taken worldwide by residents of different areas versus their official population in 2008.

*City*	*Population (mln)*	*Photographs taken*	*Photographs per 1000 residents*
*New York City*	8.36	1,026,199	122.75
*London*	7.81	1,151,799	147.48
*Paris*	2.23	534,092	239.50
*San Francisco*	0.81	851,425	1,051.14
*Washington DC*	0.59	525,313	890.36
*Barcelona*	1.62	255,038	157.43
*Chicago*	2.85	412,246	144.65
*Los Angeles*	3.83	289,810	75.67
*Rome*	2.71	126,011	46.50
*Berlin*	3.43	182,325	53.16
*Rest of EU*	4,82.61	8,637,148	17.90
*Rest of the US*	2,87.61	7,347,003	25.55
*Rest of the world*	5,905.14	6,877,894	1.16

We found that among ten most photographed places in the world there are five US and five European cities. Since the raw dataset does not contain information about user home locations, which is important to distinguish between a resident and a tourist, we assigned home locations to users following the procedure described in our previous paper [21]: residents of a certain city are considered to be those users who have the highest activity (expressed as the number of photographs taken) in that city for the longest timespan (calculated as the number of days between the first and the last photograph taken there) [21, 26, 27]. In the resulting dataset, we identified home location for over 40,000 users, who took over 4.4 million photographs in ten cities considered (over 83% of the photograph numbers shown in Table 1); we then consider them as residents in their home city and as tourist in every other city they appear. Moreover, we distinguish between foreign and domestic tourists where the latter are tourists visiting some city in their home country, while the former are traveling abroad. As the relationship between the number of users identified as residents and the number of photographs is linear to a high degree (*R*^2^ ≅ 0.85) [21], the number of photographs taken can be used as a proxy for city attractiveness. We make the processed dataset and some of the scripts used in the further analysis available for download (see the data availability statement) [28].

## Temporal distribution

**Fig 1:**
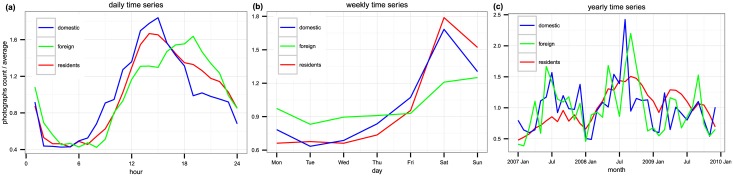
Temporal distribution of activity in New York city, showing the typical daily (a) and weekly (b) patterns and month by month activity (c) during our data collection period.

Rather than focusing only on aggregated urban attractiveness as done in [21], in this paper we focus on temporal aspects where temporal distribution is studied at three different scales: daily, weekly and annual ones, distinguishing among residents, domestic and foreign visitors. Fig 1 shows average daily and weekly patterns for these categories along with monthly aggregate numbers of photographs in New York, while S1 Fig shows the same for all cities. Looking at the apparent patterns qualitatively first, we observe that US cities residents’ behavior is similar to the domestic visitors’ behavior, while in EU cities their behavior is more similar to foreign visitors no matter the temporal scale. Looking at the differences among different time scales, daily scale shows the general behavior of the three categories (i.e., residents, domestic visitors and foreign visitors) on an average day. Apart from the similarity between categories mentioned above that we find on all three scales, what is also interesting at this scale is the general trend that in all cities we have a peak between 12.00 and 18.00. Moreover, it is interesting that there is a peak at 1.00 in the night. It is not only for visitors, but also for residents, particularly elevated in Barcelona, Rome, Chicago and Los Angeles. Moreover, for the weekly scale we can conclude that in all cities and for all categories of users the most active day is Saturday, followed by Sunday, except for foreign tourists visiting New York City, San Francisco and Paris and for domestic visitors in Barcelona, where the most active day is Sunday. Overall, the trend for foreigners is much more flat compared to residents and domestic visitors, meaning that their behavior does not change substantially during weekends and weekdays. This could be explained as they traveled longer distances to come to the city and want to experience as much as they can during their visit. It could also mean that Flickr dataset captures more of the activity of foreign visitors who came for their leisure time than of those who came for business. Finally, the yearly scale shows how behavior of different types of users changes on a month-to-month basis, with peaks of varying amplitude during the summer months in most cities especially in the case of tourists, corresponding to the yearly variation of touristic activity and photographs opportunities as well.

**Fig 2:**
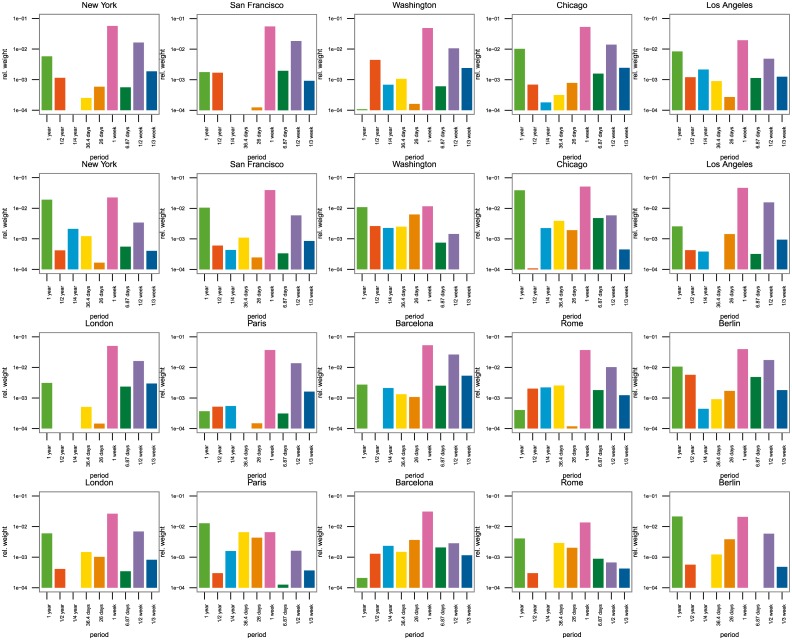
Relative importance of periodicities from the power spectrum. The nine most common frequencies are shown for each city separately for residents and tourists. From top to bottom: residents in US cities, tourists in US cities, residents in European cities, tourists in European cities. Note that the y-axis scale is logarithmic.

A quantitative way to detect periodicity in a regular series of data is to inspect its power spectrum. The power spectrum is the discrete Fourier transform of the auto covariance function of the data series. The *periodogram* plots the power versus frequency, so the seasonal patterns show up as large spikes located at their frequencies [29]. In our case, the periodogram shows the most active periods in which visitors and residents take photographs denoting the periodicity of the attractiveness in the city. Applying the aforementioned process to residents’, domestic and foreign visitors’ activities in all ten cities, we found the main periodicities as shown in Fig 2, while we display plots of the power spectra in S2 Fig and the list of most important periods in S1 Table.

As expected, the most important cycles are weekly and yearly with the former being the most important for all cases except for tourists in Berlin, augmented with harmonics forming the shape of the weekly and yearly patterns. Also for most cities it is apparent that tourists have a much stronger yearly component than residents corresponding to the more seasonal nature of tourism; more generally, we see that for residents the weekly period and periods shorter than a week are relatively more important, while for visitors, longer periods are more pronounced. This is in accordance with the observations made previously on the aggregated time series and the general assumption that for residents, the weekly cycle plays a more prominent role where several harmonics with periods shorter than a week contribute, while the number of visitors is expected to have an important yearly pattern with more variation, the shape of which can be made up of the significant harmonics with periods larger than a week.

**Fig 3:**
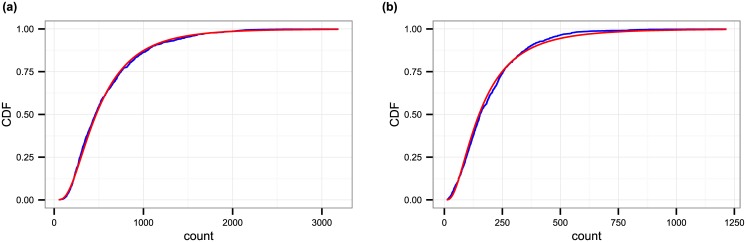
Distribution of total daily number of photographs made by New York City residents (a) and tourists (b). The blue lines correspond to the empirical data and the red lines are the fitted distributions. The fit was estimated by calculating the mean and standard deviation of the logarithm of daily photograph numbers and using a log-normal distribution with those as parameters. The associated p-values for fits are 0.244 and 0.049, respectively.

Apart from looking at sources of periodicity (i.e., regular components) in the time series, we look at the variation of photograph numbers in general and also after decomposing the time series into deterministic and random components. Looking at the distribution of raw daily photograph numbers at first, we get that the distributions can be well approximated by a log-normal distribution (note: for residents and all cities: p >0.05, i.e., we cannot rule out the null hypothesis of a log-normal distribution with 95% confidence; for tourists, p values are smaller but the distribution still fits well visually). An example for New York City is shown in Fig 3.

**Fig 4:**
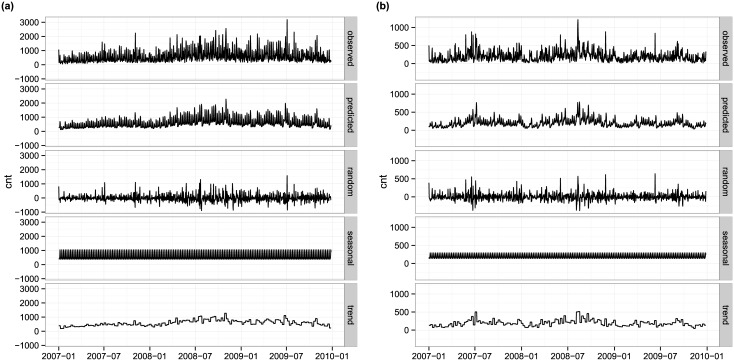
The decomposition of the time series of attractiveness in New York City considering additive noise for residents (a) and visitors (b). The first row is the observed data, the second is the deterministic part (i.e., trend and seasonal), the third the random component, while the 4^th^ and 5^th^ rows are the seasonal (cyclic) part and the trend respectively.

**Fig 5:**
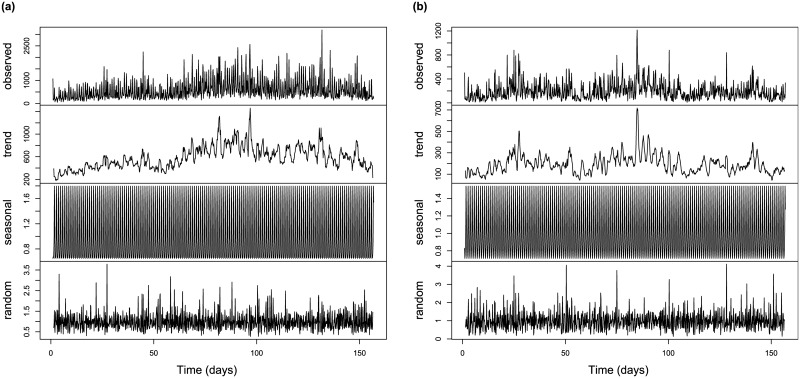
The decomposition of the time series of attractiveness in New York City considering multiplicative noise for residents (a) and visitors (b). The first row is the observed data, the second the general trend, the third the seasonality (cyclic events) and the last one is random (special events). Note that the random component here is a scaling factor which multiplies the deterministic part.

Next, we look at how time series of daily photograph numbers can be decomposed into the combination of deterministic and stochastic components and estimate the relative importance and again approximate the distribution of these. We use the following two options to decompose the attractiveness time series:

$$A_{t}=f\left(t\right)+u_{t}$$

$$A_{t}=u_{t}f\left(t\right)$$

where *A*_*t*_ denotes temporal attractiveness, *f*(*t*) is a deterministic sequence and *u*_*t*_ is the noise component, with the two options being models with additive and multiplicative noise, respectively. Further, the deterministic sequence consists of two parts: trend (d_t_) and seasonality (s_t_): *f*(*t*) = *s*_*t*_ × *d*_*t*_, while the stochastic sequence is representative of the random variations. To carry out the decomposition, we need to specify the periodicity in the seasonal component; based on the power spectrum, we chose this to be one week. Illustration of this procedure for New York City is shown in Figs 4 and 5 for the additive and multiplicative cases respectively.

We consider the noise as the most interesting component because it may represent the special events in a city as deviations from the regular rhythm of activities. To quantify the importance of the noise, we report average normalized residuals in S1 Table, computed as $<\varepsilon >\;=\;<\frac{|u_{t}|}{\frac{\left(A_{t}+f(t)\right)}{2}}>$ in the case of additive noise and as <*ε* > = < |*u*_*t*_ − 1|> for multiplicative noise, giving the relative importance or weight of noise when compared to the real activity and the deterministic component. Looking at values in Table 2, we see that they are relatively large, the noise is comparable to about 30%–60% of activity on average, while the trend shows that noise decreases as total activity increases (in accordance with what we expect based on the central limit theorem).

**Table 2:** Average normalized noise for different types of decomposition and different models.

	*Residents*		*Visitors*	
*City*	*Additive noise*	*Multiplicative noise*	*Additive noise*	*Multiplicative noise*
*New York City*	0.3026359	0.3062597	0.3894293	0.3597991
*London*	0.3178385	0.3146551	0.4035543	0.3880250
*Paris*	0.3626344	0.3537219	0.4374607	0.3916178
*San Francisco*	0.3270483	0.3283090	0.4709700	0.4354612
*Berlin*	0.4953906	0.4751745	0.5729734	0.5173991
*Washington*	0.4761626	0.4550284	0.5677503	0.5191039
*Barcelona*	0.4322980	0.4200787	0.6452356	0.6013710
*Rome*	0.5695353	0.5461870	0.6448882	0.5690575
*Chicago*	0.4131704	0.4029115	0.6433112	0.5797068
*Los Angeles*	0.5194782	0.5004668	0.5874662	0.5491432

**Fig 6:**
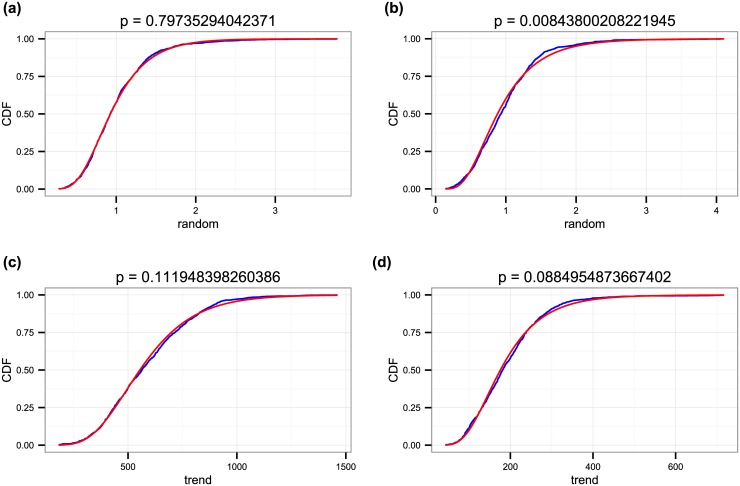
Distribution of the random component (a,b) and trend (c,d) for residents (a,c) and tourists (b,d) in New York City. Blue lines are the empirical data and red lines are the fitted distributions. With the exception of the random component for tourists, all can be considered a log-normal distribution according to the formal Kolmogorov-Smirnov test.

Looking at the distribution of individual components, we get that in the case of a multiplicative noise, the random component can also be described with a log-normal distribution for residents and gives a good approximation in the case of tourists for most cities. The trend distribution can also be approximated by a log-normal distribution; here, it gives a good fit in the case of tourists in London, Barcelona, Chicago and Los Angeles and residents in New York, while for the other cases the null hypothesis can be formally rejected, but the distribution is still rather close to a log-normal (see Fig 6). To quantify to quality of fits, we give the associated p-values for all cases in S2 Table.

We also extracted a list of what we called “outliers”, for each city. Particularly interesting in this sense are the outliers relative to the random variations, because it is supposed to be able to retrace special events. It is generally more interesting in this case detecting the activity of residents. Regarding the American cities, we noted that, in fact, from the outliers of the residents it is possible to retrace the main important events for the population. In particular, in each city (except Los Angeles) there is a trace of the 4^th^ July. This seems to be the most photographed event for the American people. Furthermore, in New York City, we found outliers related to the Thanksgiving Day for each year. For example, we found an interesting outlier for residents in Barcelona on 27 May 2009, when the soccer team won the Champions League.

## Spatio-temporal attractiveness

Having examined temporal patterns in the data, we now proceed with examining if there is an interrelation between spatial and temporal patterns of photographic activity. Similarly to [30], we use the dilatation index and dilatation coefficient to characterize if urban attractiveness is distributed equally across the city or rather concentrated in only a few of its major areas. This gives us an idea of collective behavior on the attractiveness and allows us to understand and measure the pulse of the dynamic urban system. We start with considering the “Venables index”, defined as:

$$V\left(t\right)=\sum_{i<j}^{}s_{i}\left(t\right)s_{j}\left(t\right)d_{i,j}$$

where, in our case: *s*_*i*_(*t*) denotes the number of shared photographs in cell *i* at time *t* and *d*_*i*,*j*_ is the distance between cells *i* and *j*. When all the activities are concentrated only around one single point, the value of *V* is equal to its minimum, zero. By normalizing *V* with the densities of activity in each cell, we obtain the weighted average distance *D*_*v*_ (the “Venables distance”):

$$D_{v}\left(t\right)=\frac{\sum_{i<j}^{}s_{i}\left(t\right)s_{j}\left(t\right)d_{i,j}}{\sum_{i<j}^{}s_{i}\left(t\right)s_{j}\left(t\right)}$$

**Fig 7:**
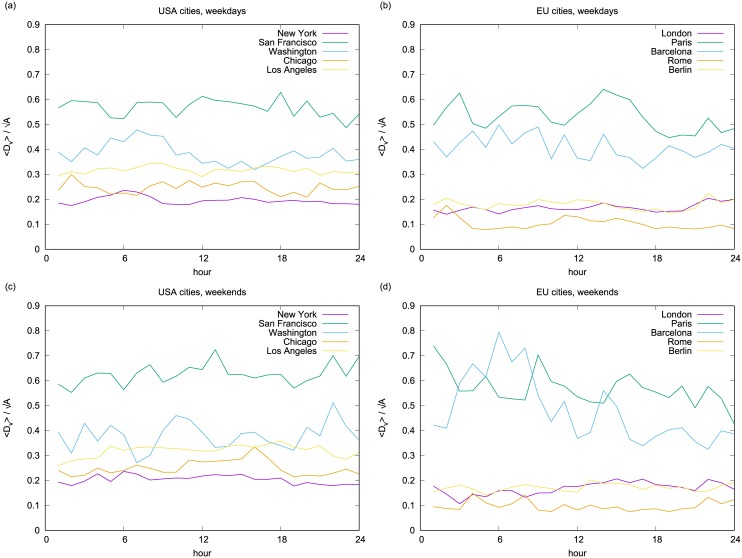
Dilatation of attractiveness. These figures show the dilatation attractiveness, evaluated with the explained method, for the 10 most photographed cities, considering the American cities and the Europeans ones in an average weekday (a,b) and in an average weekend day (c,d).

In a monocentric city, i.e., where attractive places are spatially “segregated”, we expect a large variation of the average distance *D*_*v*_ during the day as people converge around these during the day. For more polycentric cities, where places are spatially less separated, we expect a smaller variation of *D*_*v*_ than the one observed for monocentric cities as places are more “mixed”, i.e., people and activity need not concentrate in just one area or few areas during the day. In order to make sure that values of *D*_*v*_ are comparable among different cities, they should be normalized by the city dimension, $\sqrt{A}$ [30], giving the *dilatation attractiveness*:

$$Dil=\frac{D_{v}}{\sqrt{A}}$$

where *A* denotes city official area. We compute *Dil* for typical weekdays and weekend days in each city, as shown in Fig 7. When the value for dilatation attractiveness *Dil* is lower that means the attractiveness of the city is not equally distributed in all city areas, but it “collapses” in few places that are close together.

We can conclude that generally European cities are characterized by lower values of dilatation (i.e., lower than 0.2), with the exception of Barcelona and Paris, which, unlike more “dilated” US cities also show several peaks on weekends. The US cities are in general characterized by higher values of dilatation (i.e., 0.2–0.6), but their curves are flatter than the European ones with San Francisco being the most “dilated” US city. The most interesting difference between a weekday and a weekend pattern can be observed in a case of Barcelona in the early morning. There are several possible explanations for that—one of them being people who go out late at night are distributed more evenly in the city and not only in the center. Moreover, one can also notice an interesting invert peak of dilatation in Washington DC around 7 AM when on a weekday everyone is getting up in the morning, while on weekends everyone is still sleeping. From these curves, we can extract another feature: the dilatation index, as the ratio between the maximum and the minimum *D*_*v*_ during the day that gives a measure of the maximum spatial spread of high density locations [30]:

$$\mu =\frac{\max_{t}\left(D_{v}\left(t\right)\right)}{\min_{t}\left(D_{v}\left(t\right)\right)}$$

In Table 3 we display the computed dilatation index values (*μ*) of the cities. When comparing values of dilatation indices for the US and European cities, we can notice a tendency for former ones to mostly have a smaller dilatation index that the latter ones. Lower values of parameter *μ* denote that the average distance *D*_*v*_ stays approximately the same throughout the day, meaning that no matter which hour of the day it is, the spatial spread of the high density locations does not change significantly. When places of activity are more entangled, then we talk about more “mixed” cities. In the opposite case of large values of *μ*, the spatial organization of the different high-density locations changes significantly along the day.

**Table 3:** Cities ranked by the dilatation index, distinguishing average weekday and average weekend day.

*Weekdays*			*Weekends*		
1	Los Angeles	1.22	1	San Francisco	1.31
2	San Francisco	1.24	2	New York City	1.33
3	New York City	1.32	3	Los Angeles	1.38
4	Paris	1.33	4	Berlin	1.44
5	Berlin	1.36	5	Chicago	1.56
6	Chicago	1.41	6	Paris	1.74
7	Washington DC	1.49	7	Washington DC	1.89
8	London	1.52	8	London	1.93
9	Barcelona	1.54	9	Barcelona	2.01
10	Rome	1.71	10	Rome	2.44

## Summary and Conclusions

The common reason why people travel and visit new cities, as either domestic or foreign visitors, is to experience new places they feel attracted to. A very direct measure of this attractiveness is the number of publicly shared photographs that people take in cities all around the world. However, a measure of urban attractiveness is not static as it evolves over time, sometimes resulting in considerable variations over the course of a year or even over a single week or a day. In this study we thus conducted an analysis of these temporal variations, providing and comparing longitudinal characteristic for urban attractiveness patterns. Moreover, spatial variations of attractiveness cannot be separated from temporal variations. The movement itself usually is not the goal of an individual, but an intermediate step that is accomplished in a given period of TIME to reach SPACE where the individual can take advantages of interest (attractiveness). Following this logic, in our study we have analyzed not only the daily temporal dynamics (necessary for moving between two places in the territory), but also the periodicity in weeks and months. Extrapolating this information can be very useful for studying attractiveness in general, but especially for the estimation of large flows of users and for preparing to manage them, for example during special events or particular situations.

The novelty of our study is in the kind of data considered: geo-tagged photographs shared using the most popular photograph sharing platforms (e.g., Flickr). Different sources of big data have been used in planning science for a long time now (e.g., mobile phone records for the transportation planning), but photographs can give us something more useful for urbanism in general—information about what people consider important and attractive, for any reasons, in the city. For top ten most photographed cities in the world (i.e., five US cities and five EU ones), we showed the general temporal distribution comprehending the daily, weekly and month-by-month variation to detect behavior of residents, domestic and foreign visitors. Results of our analysis on temporal distribution showed that for US cities visitors’ behavior in general is more similar to domestic visitors’ behavior, while for EU cities, visitors’ general behavior is more similar to the foreign ones. We also looked at the Fourier spectrum to identify the most important periodicity of attractiveness. Periodicity is what happens to the flow within any given period or season, and is related to shifts in arrivals according to the day of the week and/or the week of the month. The causes of periodicity are many and varied. We did not analyze the causes, but we checked how and when periodicity is distributed in the city over the time. This is quite significant in urban science. When seasonal flow demand decreases, in fact, this has both direct and indirect effects upon city economy and wellness. What a city should do is to provide price incentives to encourage the off season traveler with discounts and other associated benefits. Knowing the periodicity of big flows could concretely help administrations, operators and users to manage transportation, organize events and choose the period of vacation.

Our results suggest that for residents, the weekly pattern is most pronounced, while for visitors the yearly pattern is more important. We considered the time series activities for residents and visitors, extracting trend, seasonality and random events. We found that on average, the random component constitutes about 30%-60% of activity (where the exact number depends on the overall volume of activity); this also means that in the case of a special event, it can be much higher. This method could be very interesting in particular for small cities, where special events are not so frequent as in metropolitan areas and thus it is possible to really take in account the impacts of them compared to the normal trend. Although in all cases we showed results only for our ten cities, the same methodology can be also applied to other cities as well.

Finally, we applied two spatio-temporal measures, as space and time cannot be considered just separated contributors: the dilatation attractiveness and the dilatation index, which give us measures of how the attractiveness is distributed across the city area, by combining spatial and temporal distribution. These measures revealed even more of differences between European and US cities. Namely, by looking at the “dilatation attractiveness” index, we found that the US cities in general have higher values of dilatation attractiveness and less variations compared to European cities. This means that the US cities are more polycentric and “mixed” cities, perhaps because of the fact that on average they are bigger than European cities. In fact, for a higher value of “dilatation attractiveness” city is more “dilated”, more active in all their space. This is also true for the second index, which is the ratio between the maximum and minimum value of “dilatation attractiveness” during the day. In conclusion, we measured and analyzed spatio-temporal configuration of attractiveness in different cities and we found important similarities and symmetries among them. This confirms previous research on urban human behavior displaying high predictability in general [3, 31].

## Supporting Information

S1 Fig Overview of activity time series. Average daily and weekly time series and monthly activity for each of the cities included in our analysis.
(PDF)

S2 Fig Power spectra of activity. Power spectrum of activity time series for each of the cities separately for residents (left column) and visitors (right column).
(PDF)

S1 Table Most important periods. List of most significant periods identified in the power spectra for each city.
(PDF)

S2 Table p-values for fitting the distribution of daily events with a log-normal distribution for the model with multiplicative noise (values under 0.05 would allow the hypothesis of a log-normal distribution to be rejected with 95% confidence, under 0.01 would allow rejection with 99% confidence, etc.; for most cases, the log-normal distribution cannot be rejected). (PDF)
