# Supplementary figures and images for: Uncovering Urban Temporal Patterns from Geo-Tagged Photography
Source: PLoS One. 2016 Dec 9;11(12):e0165753. doi: 10.1371/journal.pone.0165753 (PMC5148589; doi:10.1371/journal.pone.0165753)

Daily

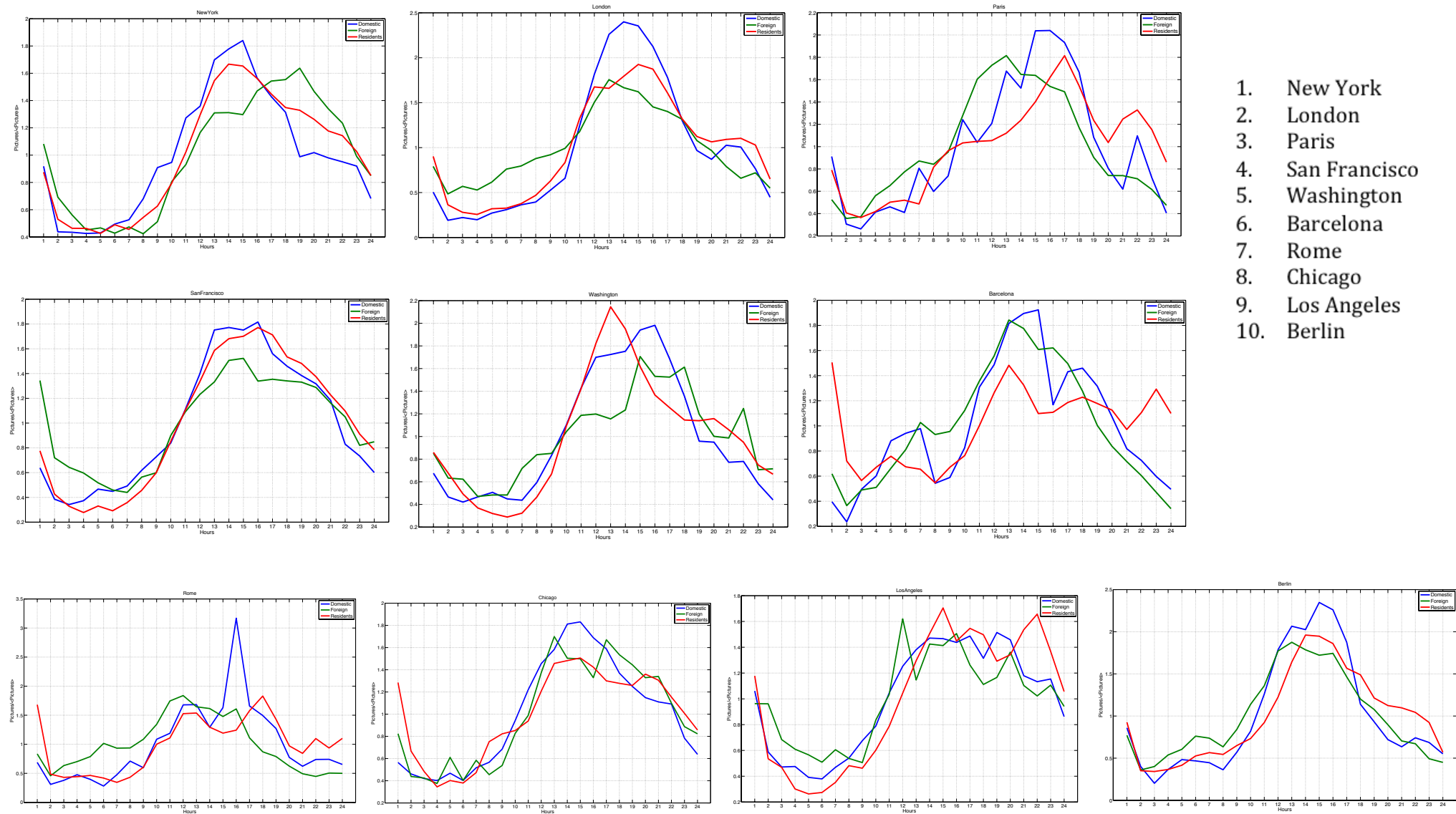

Weekly

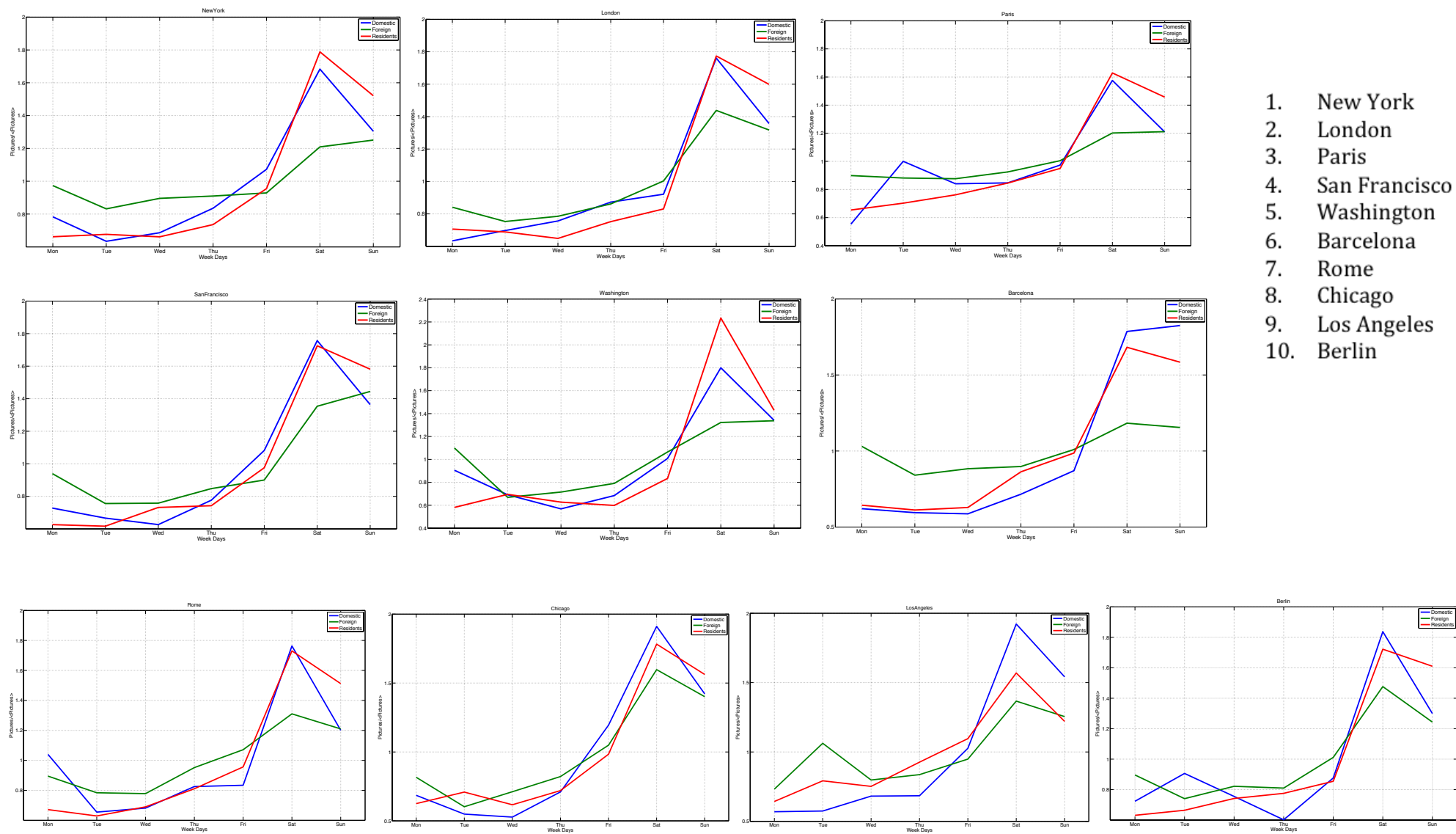

Month-by-month

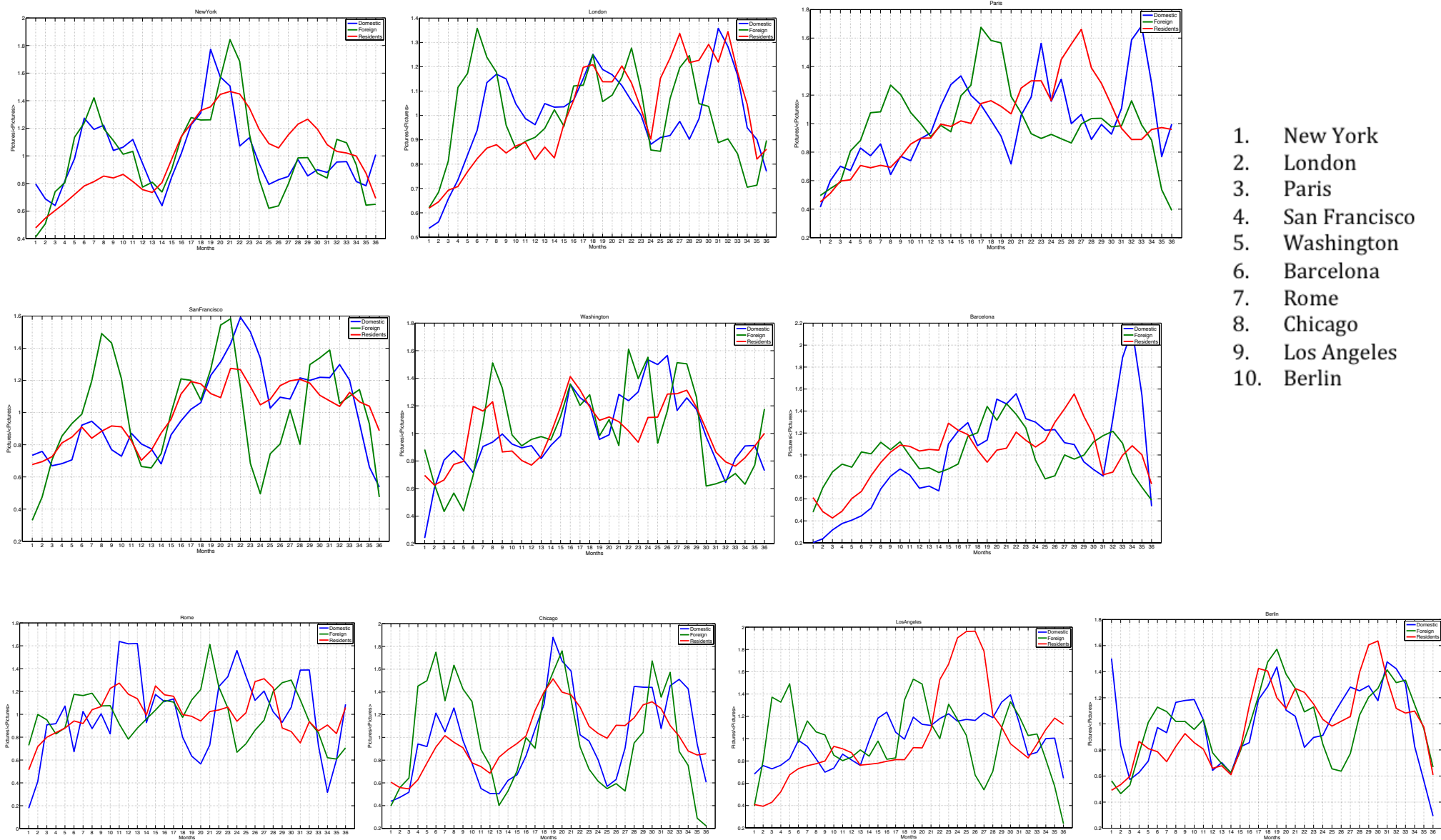

Supplement: S1 Fig — Average daily and weekly time series and monthly activity for each of the cities included in our analysis. (PDF) [file pone.0165753.s001.pdf]

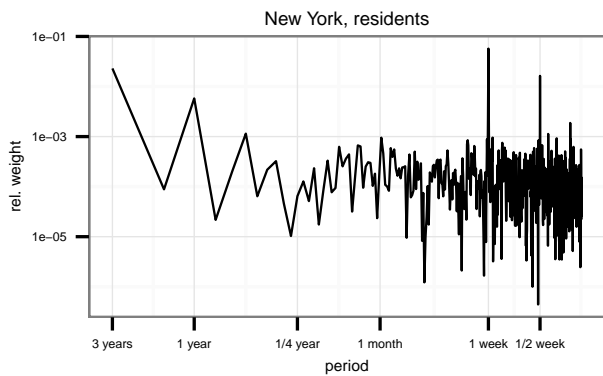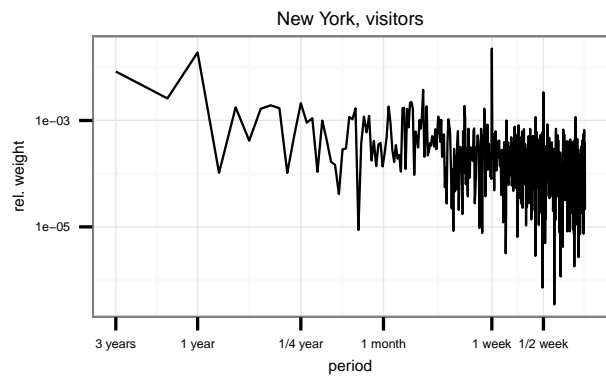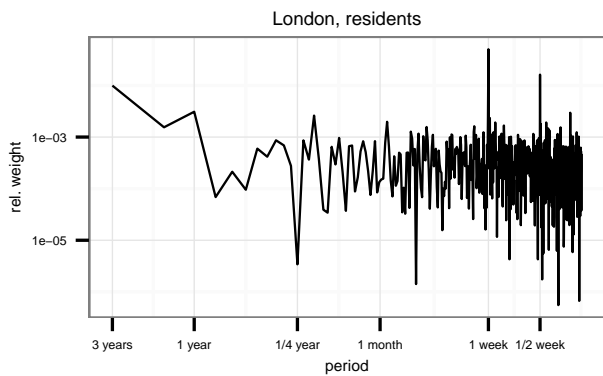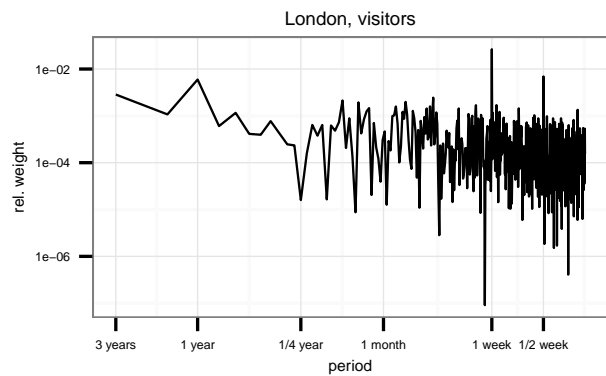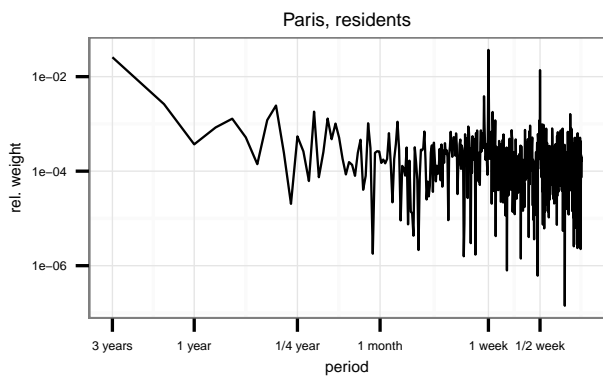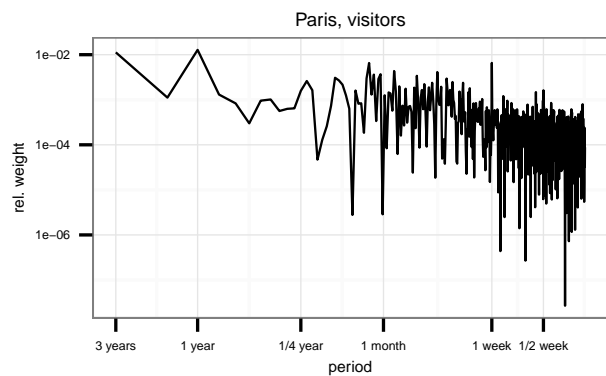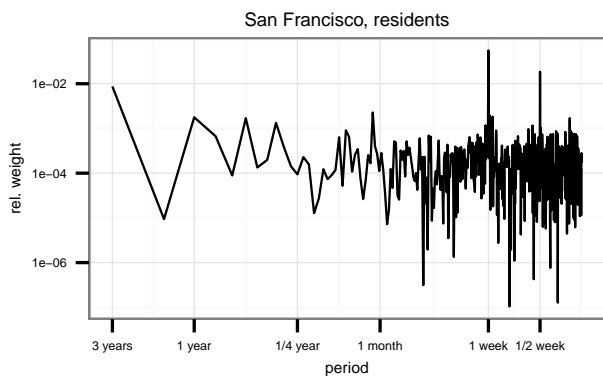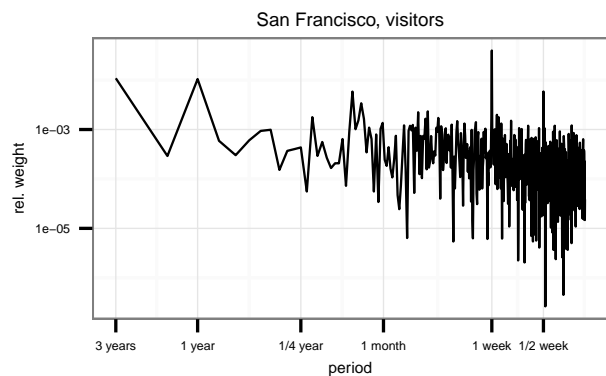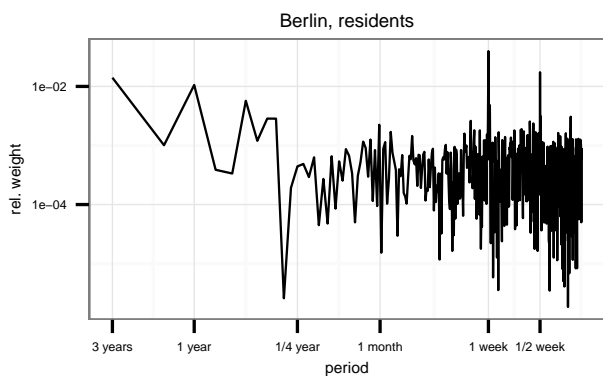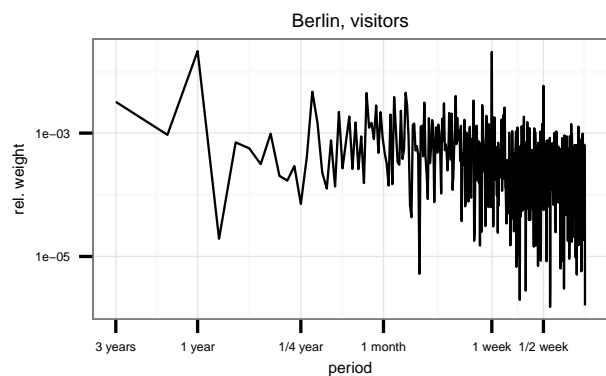

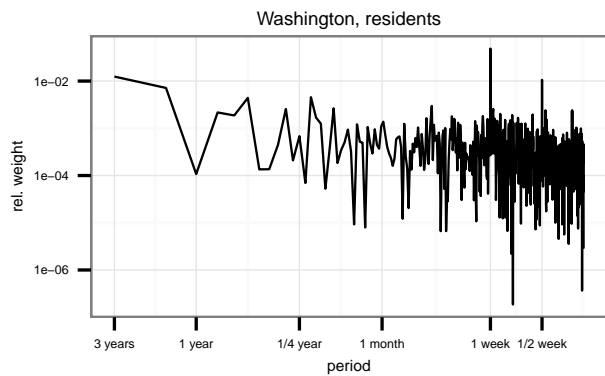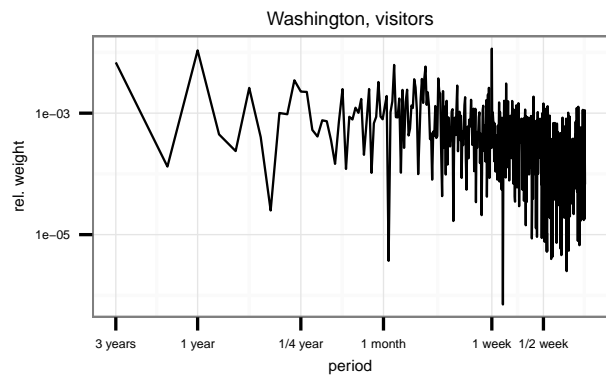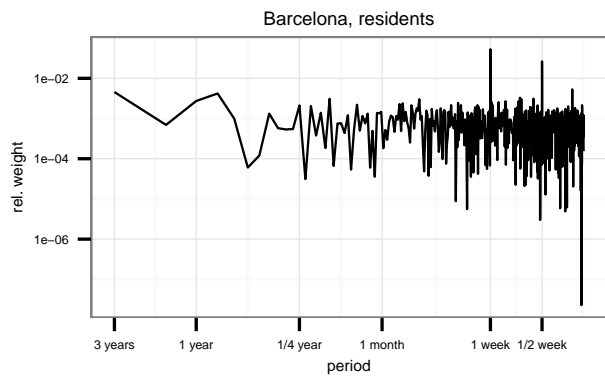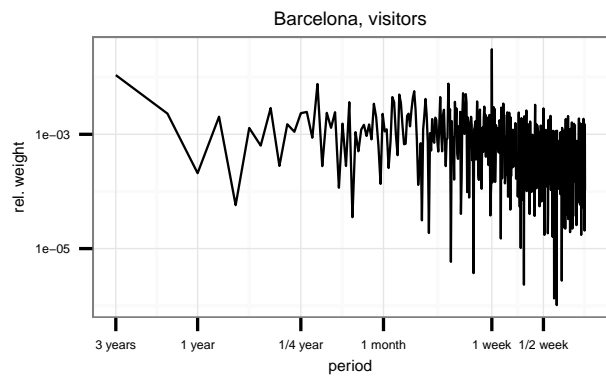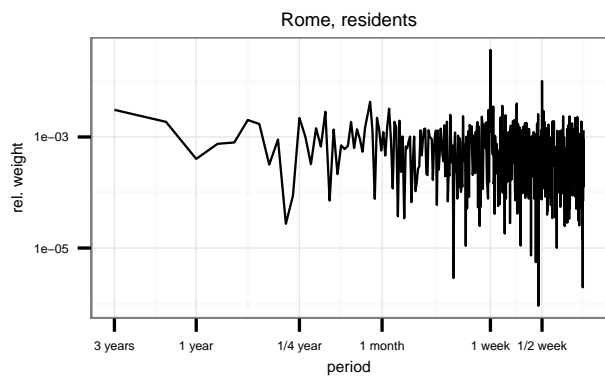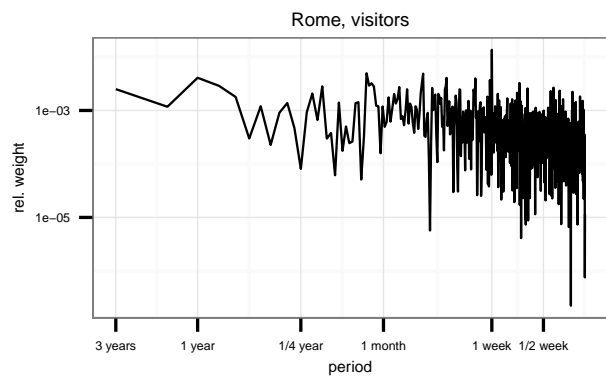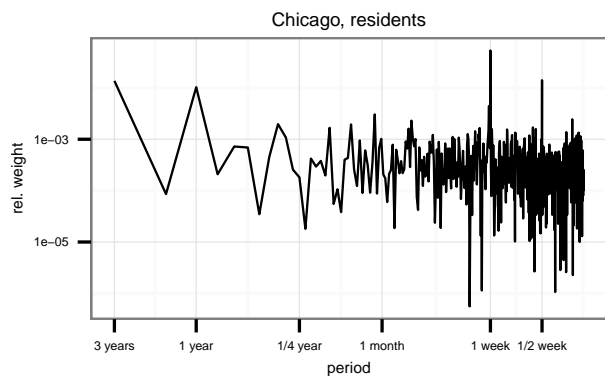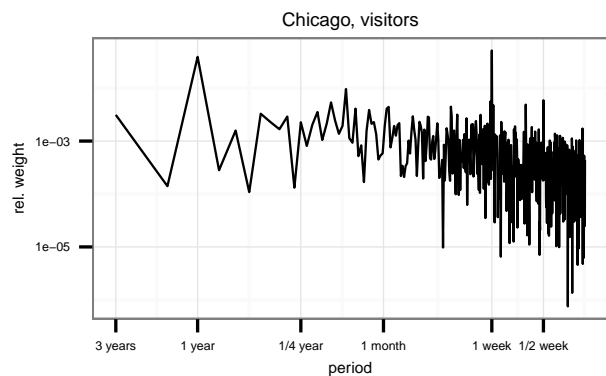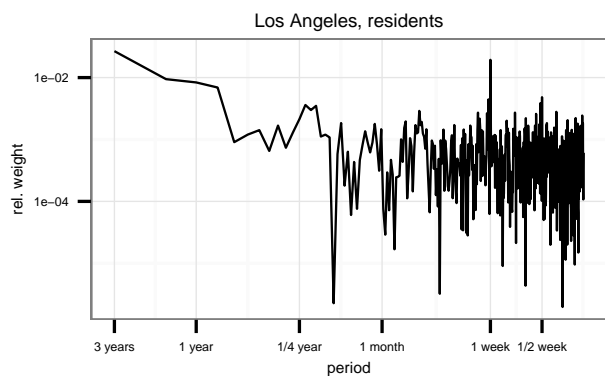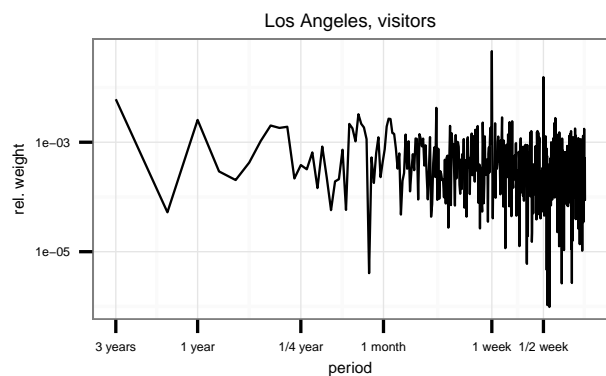

Supplement: S2 Fig — Power spectrum of activity time series for each of the cities separately for residents (left column) and visitors (right column). (PDF) [file pone.0165753.s002.pdf]
