# Supplementary material for: Uncovering Urban Temporal Patterns from Geo-Tagged Photography
Source: PLoS One. 2016 Dec 9;11(12):e0165753. doi: 10.1371/journal.pone.0165753 (PMC5148589; doi:10.1371/journal.pone.0165753)
Supplement: S1 Table — List of most significant periods identified in the power spectra for each city. (PDF) [file pone.0165753.s003.pdf]

New York, residents			New York, tourists	
rel. weight	period		rel. weight	period
5.7184E−2	7.0		2.2431E−2	7.0
2.2999E−2	1092.0		1.9014E−2	364.0
1.6306E−2	3.5		8.3217E−3	1092.0
5.7857E−3	364.0		3.7657E−3	17.613
2.1023E−3	7.045		3.3943E−3	3.5
1.8738E−3	2.333		2.5986E−3	546.0
1.2659E−3	6.955		2.2226E−3	20.604
1.1451E−3	182.0		2.1496E−3	21.0
1.1290E−3	3.138		2.1115E−3	91.0
9.5332E−4	6.618		1.9264E−3	136.5
London, residents			London, tourist	
rel. weight	period		rel. weight	period
5.0373E−2	7.0		2.6443E−2	7.0
1.6205E−2	3.5		6.9311E−3	3.5
9.9361E−3	1092.0		5.9643E−3	364.0
3.0984E−3	364.0		2.8543E−3	1092.0
2.9621E−3	2.333		2.4484E−3	15.38
2.6090E−3	72.8		2.1268E−3	52.0
2.3592E−3	7.045		1.9927E−3	22.286
2.3428E−3	6.868		1.9364E−3	42.0
1.9774E−3	27.3		1.6955E−3	6.578
1.9187E−3	6.386		1.6888E−3	8.667
Paris, residents			Paris, tourists	
rel. weight	period		rel. weight	period
3.6830E−2	7.0		1.2791E−2	364.0
2.5687E−2	1092.0		1.1244E−2	1092.0
1.3728E−2	3.5		6.5568E−3	7.0
3.8451E−3	7.429		6.5439E−3	36.4
2.6193E−3	546.0		4.3390E−3	26.0
2.5157E−3	6.955		4.1156E−3	14.56
2.4448E−3	121.333		3.6803E−3	31.2
1.8170E−3	72.8		3.6140E−3	34.125
1.7878E−3	6.618		3.3959E−3	19.158
1.6123E−3	2.333		3.0763E−3	57.474
San Francisco, residents			San Francisco, tourists	
rel. weight	period		rel. weight	period
5.5340E−2	7.0		3.9650E−2	7.0
1.8467E−2	3.5		1.0874E−2	1092.0
8.6582E−3	1092.0		1.0480E−2	364.0
2.2726E−3	33.091		5.8651E−3	3.5
1.9474E−3	6.868		5.8497E−3	45.5
1.8368E−3	6.618		3.3958E−3	40.444
1.7733E−3	364.0		2.3280E−3	16.545
1.6964E−3	182.0		2.2347E−3	18.828
1.6933E−3	2.348		1.9821E−3	6.539
1.3324E−3	121.333		1.7785E−3	78.0

Berlin, residents			Berlin, tourists	
rel. weight	period		rel. weight	period
3.9622E−2	7.0		2.1324E−2	364.0
1.7385E−2	3.5		2.0721E−2	7.0
1.4064E−2	1092.0		5.8373E−3	3.5
1.0578E−2	364.0		4.6971E−3	78.0
5.7143E−3	182.0		4.5128E−3	22.286
4.8292E−3	6.868		4.4705E−3	37.655
3.1107E−3	3.467		3.9989E−3	11.375
3.0707E−3	2.318		3.8456E−3	26.0
2.8535E−3	136.5		3.3676E−3	8.951
2.8497E−3	121.333		3.2108E−3	1092.0
Washington, residents			Washington, tourists	
rel. weight	period		rel. weight	period
4.8684E−2	7.0		1.1560E−2	7.0
1.2451E−2	1092.0		1.0817E−2	364.0
1.0596E−2	3.5		6.8486E−3	1092.0
7.1584E−3	546.0		6.2262E−3	26.0
4.5623E−3	78.0		5.8539E−3	17.063
4.3996E−3	182.0		3.7361E−3	14.368
2.9764E−3	15.38		3.7245E−3	17.333
2.6496E−3	57.474		3.6632E−3	17.902
2.5699E−3	6.741		3.6235E−3	19.855
2.5638E−3	109.2		3.4793E−3	99.273
Barcelona, residents			Barcelona, tourists	
rel. weight	period		rel. weight	period
5.2909E−2	7.0		3.0778E−2	7.0
2.6394E−2	3.5		1.0853E−2	1092.0
5.3238E−3	2.333		7.6951E−3	12.552
4.5616E−3	1092.0		7.6018E−3	72.8
4.2014E−3	273.0		5.6817E−3	19.855
3.2961E−3	4.707		5.2341E−3	10.302
3.1130E−3	3.928		5.0470E−3	9.927
3.0969E−3	60.667		4.9569E−3	24.267
3.0551E−3	4.627		4.4848E−3	26.634
3.0173E−3	18.2		4.4809E−3	14.0
Rome, residents			Rome, tourists	
rel. weight	period		rel. weight	period
3.7054E−2	7.0		1.3562E−2	7.0
1.0175E−2	3.5		4.9532E−3	37.655
4.3127E−3	35.226		4.8763E−3	17.613
4.0036E−3	4.919		4.0806E−3	364.0
3.6478E−3	5.967		4.0661E−3	12.847
3.5391E−3	6.741		3.9673E−3	8.736
3.2437E−3	27.3		3.6965E−3	7.184
3.0822E−3	1092.0		3.2634E−3	4.832
3.0665E−3	7.184		3.2610E−3	17.902
2.9502E−3	3.434		3.2226E−3	35.226

Chicago, residents			Chicago, tourists	
rel. weight	period		rel. weight	period
5.3397E−2	7.0		5.1402E−2	7.0
1.4105E−2	3.5		3.8918E−2	364.0
1.3686E−2	1092.0		9.6182E−3	49.636
1.0288E−2	364.0		5.8955E−3	3.5
4.4477E−3	7.137		5.5980E−3	7.137
3.0434E−3	33.091		5.3719E−3	60.667
2.4461E−3	2.333		4.8786E−3	4.439
2.3023E−3	20.222		4.7882E−3	6.868
1.9693E−3	121.333		4.4629E−3	12.133
1.9423E−3	45.5		4.4156E−3	28.0
Los Angeles, residents			Los Angeles, tourists	
rel. weight	period		rel. weight	period
2.6769E−2	1092.0		4.6177E−2	7.0
1.9361E−2	7.0		1.5473E−2	3.5
9.4411E−3	546.0		6.1037E−3	1092.0
8.3510E−3	364.0		4.2507E−3	14.757
6.9111E−3	273.0		3.2476E−3	42.0
4.8234E−3	3.5		2.8465E−3	6.101
4.4514E−3	7.184		2.7669E−3	2.984
3.8590E−3	3.58		2.6945E−3	28.0
3.6129E−3	84.0		2.6542E−3	27.3
3.4980E−3	72.8		2.5541E−3	364.0
