# Supplementary material for: Uncovering Urban Temporal Patterns from Geo-Tagged Photography
Source: PLoS One. 2016 Dec 9;11(12):e0165753. doi: 10.1371/journal.pone.0165753 (PMC5148589; doi:10.1371/journal.pone.0165753)
Supplement: S2 Table — (PDF) [file pone.0165753.s004.pdf]

	Raw numbers		*Random component*		*Trend*	
City	*Residents*	*Tourists*	*Residents*	*Tourists*	*Residents*	*Tourists*
New York City	0.24372	0.04895	0.79735	0.00844	0.11195	0.0885
London	0.08872	0.03826	0.2358	0.16964	0.0045	0.14844
Paris	0.78324	0.01057	0.14576	4.41E-05	0.00943	0.000294
San Francisco	0.25023	0.00566	0.62568	0.0111	0.00792	0.03389
Berlin	0.41281	0.00372	0.02391	0.00108	0.00843	0.00124
Washington DC	0.14779	0.00957	0.01697	0.0045	0.000258	0.04245
Barcelona	0.57475	0.00943	0.12524	0.000114	0.00709	0.36675
Rome	0.4739	0.00591	0.00103	0.000977	0.03103	0.00026
Chicago	0.71942	0.01429	0.49014	0.000119	0.0154	0.78193
Los Angeles	0.504	0.08066	0.15015	0.00781	0.001165	0.08973

Table 1: **p-values for fitting the distribution of daily events with a log-normal distribution for the model with multiplicative noise (values under 0.05 would allow the hypothesis of a log-normal distribution to be rejected with 95% confidence, under 0.01 would allow rejection with 99% confidence, etc.; for most cases, the log-normal distribution cannot be rejected).**
